# People of Lower Social Status Are More Sensitive to Hedonic Product Information—Electrophysiological Evidence From an ERP Study

**DOI:** 10.3389/fnhum.2019.00147

**Published:** 2019-05-15

**Authors:** Di Chen, Weiguo Qu, Yanhui Xiang, Jiaxu Zhao, Guyu Shen

**Affiliations:** ^1^Department of Psychology, Hunan Normal University, Changsha, China; ^2^Cognition and Human Behavior Key Laboratory of Hunan Province, Hunan Normal University, Changsha, China

**Keywords:** event-related potential, social status, hedonic, utilitarian, threat

## Abstract

Consumer psychology research has shown that individuals of different social statuses have distinctive purchase intentions for different products. Individuals of a high social status will simultaneously measure the symbolic status meaning and utilitarian value of a product, but they will not show strong preferences for any attributes. However, individuals of a low social status show strong purchasing tendency for hedonic products that are associated with symbolic status meaning and could satisfy their spiritual needs. This phenomenon may be due to self-threat, which caused by hedonic products. Based on the above, this study compares the cognitive processing differences of hedonic and utilitarian label products between high- and low-social-status groups by recording event related potentials (ERPs). The results showed that under the P2, P3, and LPP components, the low-social-status group elicited smaller deflections in hedonic label stimuli than in utilitarian label stimuli. The high-social-status group did not show a significant difference in these components. These results suggested that individuals with a low social status are more sensitive to hedonic product information, because high-status information contained in the hedonic label induces a sense of threat in them and generates certain negative emotions.

## Introduction

Social status either refers to rank or position and reflects a hierarchical order within a group (Dawson and Cavell, 1987). Such a hierarchy of social status exists in all social species from animals to humans (Bshary et al., 2014), and have been shown to have effects on behavior and social cognition (Blader et al., 2016; Blue et al., 2016, 2018). In the human social hierarchical structure, different social levels generate different needs and dominant abilities. For example, from an evolutionary perspective, high-status individuals pay more attention to spiritual needs such as pursuing partners and reproductive goals, while low-status individuals pay more attention to material needs such as survival and safety (Faunce, 1984; Dunning and Cohen, 1992; Sivanathan et al., 2008; Vogel and Rose, 2017).

In consumer psychology, based on differences of individual needs, previous studies have divided consumption behaviors into hedonic consumption and utilitarian consumption: Hedonic consumption emphasizes both subjective and intangible attributes, such as “the multisensory, fantasy, and emotional aspects of consumers’ interactions with products”, and supports individuals in pursuing self-development, self-expression, and uniqueness of consumption (Stephens et al., 2007; Stephens et al., 2012). Utilitarian consumption emphasizes the objective and tangible attributes of a product (Kramer and Yoon, 2007), and transfers practical benefits and long-term use value to consumers (Tully et al., 2015; Weidman and Dunn, 2017). Based on this division, this study hypothesized that individuals with a high social status are more interested in hedonic consumption, while individuals with a low social status are more interested in utilitarian consumption. However, previous studies that addressed the relationship of social status and consumer behavior have shown that high-social-status groups will simultaneously measure both the status symbol and utilitarian value of products when purchasing products, but they do not show strong preferences for specific attributes. Meanwhile, low-social-status groups show stronger purchasing tendency for hedonic products, which may contain a symbolic status meaning and thus satisfy their spiritual needs (Bhattacharya, 2012; Zhao et al., 2018). These effects may explain that high-social-status people who have achieved an advantage in life and gained wealth (which leads to a higher self-conceptual integrity), do not require external factors to protect their self-concept. Therefore, they do not show strong preferences for hedonic products or utilitarian products. However, for the low-social-status individuals, the lower social status will induce a threat of their self-concept; therefore, they are more inclined to conceal or offset their low social status by purchasing hedonic products (Sivanathan and Pettit, 2010; Bhattacharya, 2012).

Although individuals with either a high or low social status can obtain happiness through hedonic consumption, the practical significance of their consumer behaviors differs. High-social-status groups are free to choose any types of consumption without being restricted by resources and can even easily obtain products with both hedonic and utilitarian attributes. However, for low-social-status groups, the symbolic status of hedonic products would constantly remind them that they are living within a comparatively low social level and have access to fewer resources. Their lives are subject to greater constraint and uncertainty and therefore, they can only purchase utilitarian products (Kraus et al., 2012). Low-social-status groups are forced to choose utilitarian consumption and therefore prefer to resolve the conflict between the status quo and self-ideal by obtaining hedonic products to overcome the real threat. A similar conclusion has been drawn in a study of overconsumption: an important reason for individuals to consume in advance is that they wish to buy high-value products to achieve self-affirmation and to resist peer threats (Braun and Wicklund, 1989; Miller and Cohen, 2001; Ledgerwood et al., 2010; Nelissen and Meijers, 2011; Khalifa and Shukla, 2017). Thus, threat is a key factor that affects individual decision-making and behavior (Cohen and Sherman, 2014; Wiebenga, 2015), and also impacts individual consumption behaviors. Self-threat is the central factor that causes individuals with a low social status to prefer products that can help them to spiritually improve their status (Rucker and Galinsky, 2009; Wilcox et al., 2009; Neuberg et al., 2011; Rucker et al., 2012; Zhao et al., 2018).

Previous studies have shown that human cognitive systems respond more quickly to negative information such as negative emotional stimuli and events (Cacioppo et al., 1999; Delplanque et al., 2004, 2005; Huang and Luo, 2006; Meng et al., 2012). These cognitive systems are particularly sensitive to self-threatening stimuli (Macleod and Mathews, 1988; Mogg et al., 1998; Bar-Haim et al., 2007). For example, individuals exert attention cognitive bias toward high-status faces (Dalmaso et al., 2014) and angry expression pictures (Ohman and Mineka, 2001).

In an ERP study, Yuan et al. (2007) showed that, compared to moderately negative stimuli and neutral stimuli, individuals are more sensitive to extremely negative stimuli (such as scenes of car accidents and photos of injuries that involve bleeding), and such stimuli will be treated with priority during information processing. This can be explained by the theory of evolution, that individuals can inadvertently process a threatening stimulus based on instinct during early cognitive processing (Huang and Luo, 2006; Franken et al., 2008). Based on the above research, we hypothesized that hedonic product information will pose a self-threat to low-social-status group. Consequently, during cognitive processing in the brain, compared to utilitarian product information, the low-social-status group is more sensitive to hedonic product information. They unconsciously process this type of information and handle it specifically. However, this phenomenon does not exist in high-social-status groups.

The present study explored the cognitive process differences for hedonic and utilitarian label products between high- and low-social-status groups by recording event related potentials (ERPs). According to previous studies, P2 is an important indicator during the early attention processing stage, and the maximum value typically appears within a 100–250 ms time-window (Stahl et al., 2010). The rapid activation of P2 often represents a quick extraction of typical stimulation features (Thorpe et al., 1996). A smaller P2 amplitude can predict faster detection of stimulus-related features (Yuan et al., 2007), and automated attention processing often induces smaller P2 amplitudes (Hansen and Hansen, 1988; Li et al., 2005). Based on this we hypothesized that low-social-status individuals will induced a smaller P2 amplitude for hedonic product stimuli than for utilitarian product stimuli. The reason for this is the automatic retrieval of individuals for status threatening stimuli under the conditions of low social status; however, this difference does not exist in high-social-status individuals. P3 represents the individual’s cognitive evaluation of the significance of a particular stimulus (Ito et al., 1998; Huang and Luo, 2006) and behavioral inhibition processes (Liu et al., 2015; Morsel et al., 2017). The maximum value typically appears in 300–450 ms time-window (Hajcak et al., 2010). Previous studies reported that individuals inhibited behavioral responses through the results of cognitive evaluation, and a higher degree of inhibition would decrease P3 amplitudes (Smith et al., 2008; Liu et al., 2015; Morsel et al., 2017). This result is particularly evident in implicit experiments. For example, in an implicit emotion experiment, negative stimuli result in smaller P3 amplitudes compared to neutral stimuli, because negative stimuli are more likely to attract individuals to focus on the available resources. However, emotional valence is task-irrelevant information in an implicit emotion experiment, and when emotional stimuli appear, the higher degree of inhibition is accompanied by a lower P3 amplitude (Yuan et al., 2007). Based on these results, this study assumed that in the Oddball implicit experimental paradigm, compared to utilitarian product labels, low-social-status individuals would show lower P3 amplitudes for hedonic product labels. However, these lower P3 amplitudes in both labels of high-social-status individuals do not show this difference. Because hedonic labels and utilitarian labels are task-irrelevant stimuli, participants will restrain them. Hedonic labels will pose a self-threat to the individual and will thus be more attractive and elicit lower P3 amplitudes. LPP is an important index with which to measure the level of late emotional arousal, and the maximum value generally appears after stimulus presentation for 400 ms (Hajcak and Nieuwenhuis, 2006; Foti and Hajcak, 2009). A number of studies have shown that LPP is an important index to measure emotional regulation, and more negative emotions after adjustment will lead to lower LPP amplitudes (Hajcak and Nieuwenhuis, 2006; Moser et al., 2006; Krompinger et al., 2008; Moser et al., 2009, 2010; Thiruchselvam et al., 2011; Mastria et al., 2017). Based on these results, we hypothesized that individuals with a low social status will have more negative LPP amplitudes for hedonic product information than for utilitarian product labels, while high-social-status individuals will not show this difference. In summary, the amplitudes of P2, P3, and LPP are reported in this study to explore the processing differences for different types of product information of different social statuses.

This study was conducted using the tristimulus Oddball paradigm (Wei et al., 2002; Polich, 2007; Jaušovec and Jaušovec, 2008; Yuan et al., 2010). The stimuli were divided into standard stimuli and deviant stimuli, and deviant stimuli were further divided into target stimuli and distracter stimuli. Participants were asked to perform a simple keystroke in response to a target stimulus. The real purpose of the experiment was concealed by the keystroke response to the target stimuli. The expected effect of the keystroke response to the deviant stimuli that needed to be measured was avoided.

## Materials and Methods

### Subjects

This study was conducted using a mixed experimental design of three social statuses (high status vs. low status vs. control; between-subjects) × two trial types (hedonic labels vs. utilitarian labels; within-subject). A total of 45 students participated in this experiment: 23 females and 22 males (mean age = 21.02 years, *SD* = 4.98). Due to the failure of social status perception priming (two samples) and procedural problems (two samples), four invalid data points were removed; therefore, the final sample size was 41, including 14 high-status people (seven females and seven males), 16 low-status people (nine females and seven males), and 11 controls (six females and five males). Prior to testing, each participant signed an informed consent form. The Ethics Committee of the authors’ institution approved this study.

### Stimuli

This study used the Oddball paradigm to explore differences in cognitive processing between different product labels in both high- and low-social-status groups. In reference to the paradigms used on previous research (Wei et al., 2002; Polich, 2007; Jaušovec and Jaušovec, 2008; Yuan et al., 2010; Lu et al., 2017; Sokhadze et al., 2009), the stimuli included large circles (standard stimuli), small circles (target stimuli), hedonic labels (distracter stimuli), utilitarian labels (distracter stimuli), and irrelevant labels (distracter stimuli). The hedonic label, utilitarian label and irrelevant label materials were selected from an existing study (Xiang et al., unpublished). At the same time, to avoid the influence of meaningful words on brain region activation, irrelevant labels were selected for non-meaningful words in combination with common Chinese characters. Irrelevant labels were added to prevent routine responses of the subjects to text labels, but they were not included in the data analysis. The experimental text materials were as follows:

Hedonic labels: 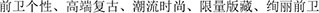


Utilitarian labels: 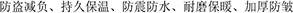


Irrelevant labels: 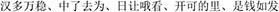


The diameter of the big circle was 4.8 cm (2.8° angle) and the diameter of the small circle was 4.2 cm (2.4° angle), the three groups of four characters had a text label size of 4 cm × 1 cm (1.7 × 0.6° angle).

### Procedures

#### Status Prime

In the beginning, to control the influence of real status and other unrelated variables, the subjects were randomly assigned to a high-social-status, low-social-status, and a control group. In reference to previous studies (Griskevicius et al., 2010; Hill et al., 2015), situational materials were used to prime high- or low-social-status groups. The high-status group read a short story intending to elicit high-status motives. In the story, participants imagined graduating from college, being admitted to a large company, which offered great working conditions, a good welfare system, and high salary. With increasing income, participants gradually entered the upper class. In the low-status group, the story was the reversed, their work opportunities, treatment, living environment, friends, and financial status were all in contrast to those described in the high-status story. After reading the respective story, participants of both groups were asked to answer the following question: “Imagine you are the protagonist in the story, to what extent do you feel you are important at the moment?” The responses were classified according to a 7-point scale ranging from 1 (not important at all) to 7 (very important). The significance levels of different in status scores between both groups were used to confirm whether the two status were successfully initiated. The control group read irrelevant material, and to avoid insinuation, control subjects were not asked any status questions.

#### Experiment

**Figure 1:**
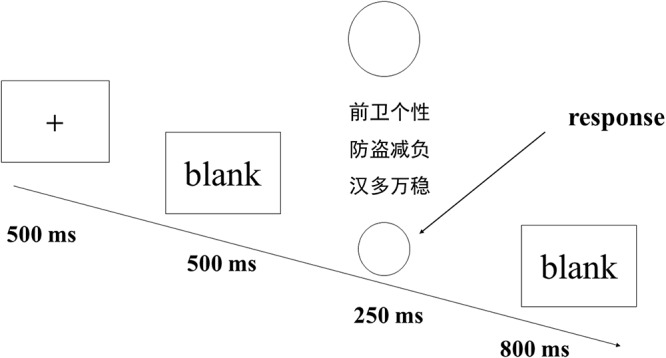
Schematic diagram of the experiment.

In the Oddball paradigm, the big circle was presented 600 times (60%), and the small circle was presented 100 times (10%). Moreover, the three sets of distractor stimulus were presented 100 times each (30%). Subjects were instructed to press the “J” key if the small circle was presented, while no response was required for other stimulus. The formal experiment was divided into four blocks, and within each block, the trials were presented randomly. In the formal experiment, each trial started with a masking stimulus “+,” which appeared in the middle of the screen for about 500 ms. Subsequently, a blank screen was presented for 500 ms, which was followed by the presentation of one of the five types of stimuli for 250 ms, and then, a blank screen was presented for 800 ms (see Figure 1). To familiarize participants with the task, the experiment was preceded by 30 practice trials, which were not repeated in the formal experiment.

#### ERP Recording and Analysis

The electroencephalography (EEG) was recorded from 64 scalp sites (Brain Products, Munich, Germany) with Ag/AgC1 electrodes. Each channel was referenced online to FCz, and two electrodes were placed at the infraorbital and supraorbital regions of the right eye to record the horizontal electrooculogram (EOG) to correct for eye-movements (blinks). Both the EEG and EOG were amplified using a DC ∼100 Hz band-pass filter and continuously sampled at 500 Hz per channel for off-line analysis. The interelectrode impedances were kept below 5 kΩ. The EEG data were preprocessed using EEGLAB (version 14.1.1b), which is an open source toolbox running on MATLAB (version R2014a). The data was sampled at 250 Hz, filtered using a high-pass filter at 1 Hz, and the filter pass-band ranged from 0.05 to 100 Hz. The mean values of the left and right mastoid were set as off-line references to reduce the influence of hemispherical asymmetrical reference points. The data were filtered (0.01–30 Hz), to mark the appearance of the target, and data was segmented with a 1000-ms time window. Then, the time schedule (epoch) was analyzed from pre-stimuli 200 ms to post-stimuli 800 ms. Pre-stimuli 200 ms was used as baseline, and all-time histories were used for the correction to improve the reliability of independent components (ICA). Then, the data were divided into the maximum independent component with a runica utilitarian, after manually excluding other human factors related to blink and lateral eye movement. Finally, all time schedules were averaged under different experimental conditions, and the final superposition times of each experimental condition were above 90% of the total number of corresponding conditions.

**Figure 2:**
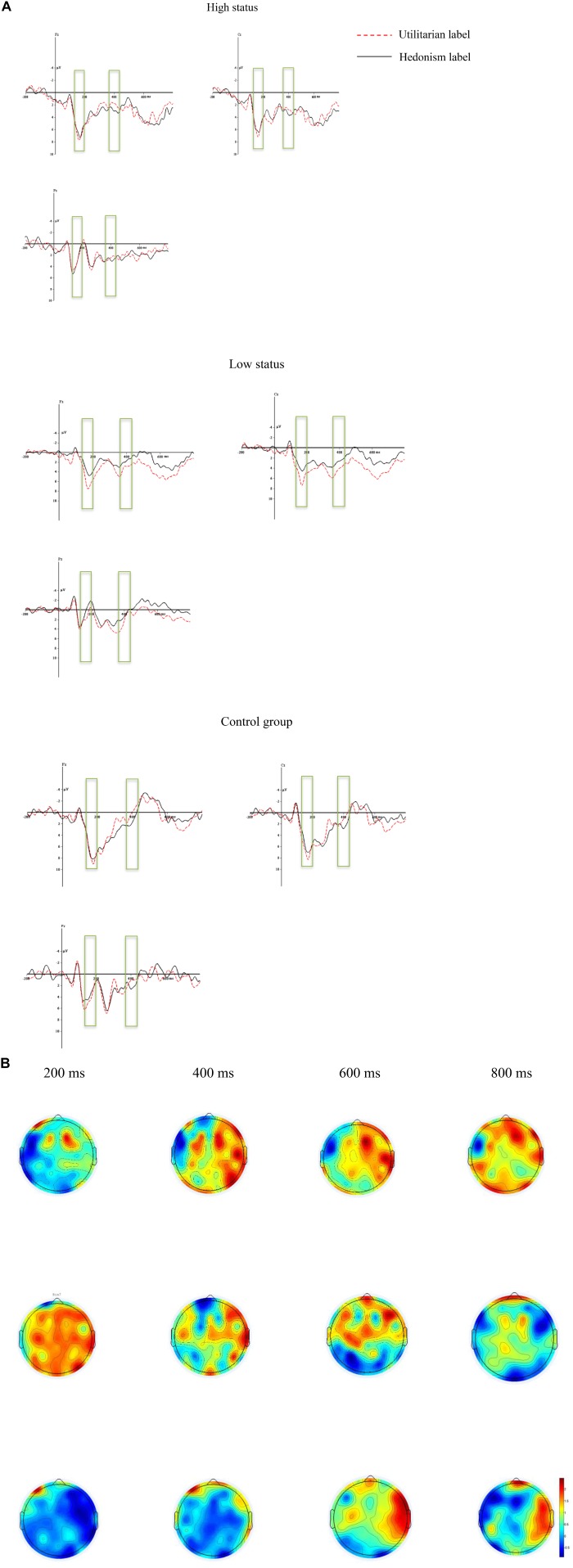
The average ERPs for different waves of the utilitarian label and the hedonic label under three different conditions **(A)**; And the scalp topographic voltage maps for difference waves of the utilitarian label - the hedonic label under three different conditions **(B)**.

The electroencephalography activity for the correct response in each label condition was overlapped and averaged separately. As shown by the ERP’s grand averaged waveforms and topographical map (see Figure 2), the ERPs elicited by both the hedonism label and the utilitarian label conditions showed prominent differences in low social status. These differences were found on central, frontal, and parietal sites. Thus, the following nine electrode sites were selected for statistical analysis: Fz, F3, F4, Cz, C3, C4, P3, P4, and Pz. The amplitudes (from baseline to peak) and peak latencies (from stimulus onset to the peak of each component) of the P2 (130–200 ms), P3 (360–450 ms), and LPP (400–800 ms) components were measured and analyzed. The above analysis was performed with SPSS 20.0, and the degrees of freedom of the F-ratio was corrected with the Greenhouse Geisser method.

## Results

### Status Priming

The results showed that, relative to the low-social-status condition (*M* = 2.21, *SD* = 0.70), the high-social-status condition (*M* = 5.50, *SD* = 0.65) elicited a “feeling of having social status” (5.50 vs. 2.21 on a 1–7 scale, *t*_(13)_ = 10.796, *P* < 0.001).

### ERP Analysis

As shown in Figure 2, P2, P3, and LPP components were elicited by different labels in all three social-status conditions.

### P2 (130–200 ms)

The mean amplitudes for a high-social status, low-social status, and the control condition were analyzed using MANOVA with different social statuses as independent variables and mean amplitudes as dependent variables. This was validated by a significant social status × label types × electrode sites [*F*_(7,136)_ = 2.390, *p* < 0.05, $\eta_{p}^{2}$ = 0.112]; this was not validated by a significant social status × label types [*F*_(2,38)_ = 0.997, *p* = 0.378, $\eta_{p}^{2}$ = 0.050]; the main effect of the status condition (high vs. low vs. control) was not significant [*F*_(1,38)_ = 1.325, *p* = 0.278, $\eta_{p}^{2}$ = 0.065]; the main effect of label type condition (hedonic labels vs. utilitarian labels) was not significant [*F*_(1,38)_ = 2.259, *p* = 0.141, $\eta_{p}^{2}$ = 0.056]; the main effect of electrode sites was significant [*F*_(2,76)_ = 23.866, *p <* 0.001, $\eta_{p}^{2}$ = 0.386]. Further analyses showed that on valid trials, only under low-social-status condition, the interactions of label types × electrode sites were significant [*F*_(3,52)_ = 3.690, *p* < 0.01, $\eta_{p}^{2}$ = 0.197]; the P2 effect of hedonic labels (*M* = 1.964 μν, *SD* = 0.894) was significantly smaller than that of utilitarian labels (*M* = 3.733 μν, *SD* = 0.764; see Table 1). Furthermore, the difference in label type in the forehead area (F3, F4, Fz) (*M*_hedonic_ = 3.216 μν, *SD* = 0.872, *M*_utilitarian_ = 5.635 μν, *SD* = 1.080) was significantly higher than the central area (C3, C4, Cz) (*M*_hedonic_ = 2.499 μν, *SD* = 1.082, *M*_utilitarian_ = 4.688 μν, *SD* = 0.928) and the parietal region (*M*_hedonic_ = 0.178 μν, *SD* = 1.562, *M*_utilitarian_ = 0.876 μν, *SD* = 0.922). The main effect of electrode sites was significant [*F*_(2,32)_ = 6.053, *p* < 0.01.$\eta_{p}^{2}$ = 0.287]. Under the condition of high social status, the interaction of label types × electrode sites were not significant [*F*_(4,47)_ = 0.266, *p* = 0.881, $\eta_{p}^{2}$ = 0.020]; the main effect of electrode sites was not significant [*F*_(4,47)_ = 0.266, *p* = 0.881, $\eta_{p}^{2}$ = 0.020]. Under the control condition, the interaction of label types × electrode sites were not significant [*F*_(3,43)_ = 0.872, *p* = 0.452, $\eta_{p}^{2}$ = 0.080]; the main effect of electrode sites was not significant [*F*_(3,43)_ = 0.872, *p* = 0.452, $\eta_{p}^{2}$ = 0.080]. Moreover, significant latency effect for valence was observed in P2 [*F*_(11,206)_ = 2.114, *p* < 0.05, $\eta_{p}^{2}$ = 0.100]. P2 latency was shorter for hedonic labels than for utilitarian labels under the condition of low social status.

**Table 1:** Mean amplitude and latencies of P2 and P3 components collapsed across the nine selected electrode sites in different conditions.

		Amplitude		Valence	
		P2	P3	P2	P3
			
		*M* (SD)	*M* (SD)	*M* (SD)	*M* (SD)
High status	Hedonism labels	4.178 (0.955)	3.172 (0.966)	159.381 (3.736)	408.254 (4.818)
	Utilitarian labels	4.268 (0.817)	2.616 (0.876)	158.095 (2.713)	403.873 (4.088)
Low status	Hedonism labels	1.964 (0.894)	2.393 (0.904)	160.708 (3.495)	400.111 (4.507)
	Utilitarian labels	3.733 (0.764)	3.640 (0.820)	163.764 (2.538)	395.264 (3.824)
Control	Hedonism labels	4.196 (1.078)	1.298 (1.090)	164.949 (4.215)	398.267 (5.701)
	Utilitarian labels	4.739 (0.922)	0.604 (0.988)	164.364 (3.061)	391.644 (4.837)

*M: mean; SD: standard deviation*.

### P3 (360–450 ms)

The mean amplitudes were analyzed for a high-social-status, low-social-status, and a control condition using MANOVA with social status as independent variable and mean amplitudes as dependent variables. This was validated by a significant social status × label types × electrode sites [*F*_(8,148)_ = 2.147, *p* < 0.05, $\eta_{p}^{2}$ = 0.102]; this was not validated by a significant social status × label types [*F*_(2,38)_ = 1.693, *p* = 0.197, $\eta_{p}^{2}$ = 0.082]; the main effect of the status condition (high vs. low vs. control) was not significant [*F*_(2,38)_ = 1.733, *p* = 0.190, $\eta_{p}^{2}$ = 0.084]; the main effect of label types condition (hedonic labels vs. utilitarian labels) was not significant [*F*_(1,38)_ = 0.000, *p* = 0.998, $\eta_{p}^{2}$ = 0.000]; the main effect of electrode sites was not significant [*F*_(2,60)_ = 0.967, *p* = 0.367, $\eta_{p}^{2}$ = 0.025]. Further analyses showed that on valid trials, only under the low social status condition, the interaction of label types × electrode sites was marginal significant [*F*_(3,43)_ = 2.708, *p* = 0.059, $\eta_{p}^{2}$ = 0.153], the P3 effect for hedonic labels (*M* = 2.393 μν, *SD* = 0.904) was significantly smaller than that of utilitarian labels (*M* = 3.640 μν, *SD* = 0.820) (see Table 1). The difference between the label type and the brain region was significant, *F*_(2,24)_ = 4.217, *p* < 0.05, $\eta_{p}^{2}$ = 0.219; the average amplitude of the utilitarian label in different brain regions is higher than that of the hedonic label, and the central region (C3, C4, Cz) (*M*_hedonic_ = 2.508 μν, *SD* = 0.827, *M*_utilitarian_ = 4.219 μν, *SD* = 0.746) showed a high difference. In the forehead area (F3, F4, Fz) (*M*_hedonic_ = 2.060 μν, *SD* = 0.945, *M*_utilitarian_ = 3.796 μν, *SD* = 1.013) and the parietal region (P3, P4, Pz) (*M*_hedonic_ = 2.611 μν, *SD* = 1.966, *M*_utilitarian_ = 2.906 μν, *SD* = 1.415). Under the condition of high status, the interaction of label types × electrode sites were not significant [*F*_(5,59)_ = 1.149, *p* = 0.344, $\eta_{p}^{2}$ = 0.081]. Under the control condition, the interaction of label types × electrode sites was not significant [*F*_(4,36)_ = 0.545, *p* = 0.684, $\eta_{p}^{2}$ = 0.052]. Moreover, no significant latency effect for valence was observed in P3 [*F*_(16,237)_ = 0.787, *p* = 0.671, $\eta_{p}^{2}$ = 0.041].

### LPP (400–800 ms)

To test the time dynamics of the change of emotional potency, it was divided into four time intervals within the 400–800 time window (400–500, 500–600, 600–700, 700–800 ms), a three (social status) × three (label types) × four (time window) MANOVA was performed on the LPP amplitude, and the main effect of time window was found to be significant [*F*_(2,74)_ = 6.851, *p* < 0.005, $\eta_{p}^{2}$ = 0.153). After multiple comparisons, the 600–700 ms window average amplitude was found to be significantly more positive than the 400–500, 500–600, and 700–800 ms time windows (all *p* < 0.005). However, there was no significant interaction between the social status groups, label type, and time windows. To further explore the results, each time window was analyzed separately.

### 400–500 ms

The mean amplitudes for the high-social status, low-social status, and the control condition were analyzed, using MANOVA with the social status as an independent variable and mean amplitudes as dependent variable. This was validated by a significant social status × label types × electrode sites [*F*_(6,117)_ = 2.197, *p* < 0.05, $\eta_{p}^{2}$ = 0.104]; this was not validated by a significant social status × label types [*F*_(2,38)_ = 1.152, *p* = 0.327, $\eta_{p}^{2}$ = 0.057]; the main effect of status condition (high vs. low vs. control) was significant [*F*_(1,39)_ = 3.613, *p* < 0.05, $\eta_{p}^{2}$ = 0.160]; the main effect of label types condition (hedonic labels vs. utilitarian labels) was not significant [*F*_(1,38)_ = 0.994, *p* = 0.425, $\eta_{p}^{2}$ = 0.025]; the main effect of electrode sites was not significant [*F*_(1,57)_ = 0.590, *p* = 0.510, $\eta_{p}^{2}$ = 0.015]. Further analyses showed that on valid trials, it was found that only under the low-social-status condition, the interaction of label types × electrode sites was significant [*F*_(2,34)_ = 4.262, *p* < 0.05, $\eta_{p}^{2}$ = 0.221]. The LPP effect for hedonic labels (*M* = 1.511 μν, *SD* = 1.287) was significantly smaller than that for utilitarian labels (*M* = 3.107 μν, *SD* = 0.975). The difference in label type in the forehead area (F3, F4, Fz) (*M*_hedonic_ = 1.553 μν, *SD* = 1.032, *M*_utilitarian_ = 4.059 μν, *SD* = 1.193) was significantly higher than in the central area (C3, C4, Cz) (*M*_hedonic_ = 1.375 μν, *SD* = 0.861, *M*_utilitarian_ = 3.591 μν, *SD* = 0.900) and the parietal region (P3, P4, Pz) (*M*_hedonic_ = 1.601 μν, *SD* = 2.358, *M*_utilitarian_ = 1.670 μν, *SD* = 1.468), *F*_(1,21)_ = 8.473, *p* < 0.005, $\eta_{p}^{2}$ = 0.361.

### 500–600 ms

This was not validated by a significant social status × label types × electrode sites [*F*_(6,119)_ = 0.768, *p* = 0.598, $\eta_{p}^{2}$ = 0.038); the main effect of status condition (high vs. low vs. control) was not significant [*F*_(1,39)_ = 2.353, *p* = 0.108, $\eta_{p}^{2}$ = 0.108]; the main effect of label types condition (hedonic labels vs. utilitarian labels) was significant [*F*_(1,39)_ = 5.567, *p* < 0.05, $\eta_{p}^{2}$ = 0.125]; the main effect of electrode sites was not significant [*F*_(1,45)_ = 0.743, *p* = 0.412, $\eta_{p}^{2}$ = 0.019].

### 600–700 ms

This was not validated by a significant social status × label types × electrode sites [*F*_(6,106)_ = 0.469, *p* = 0.817, $\eta_{p}^{2}$ = 0.024]; the main effect of status condition (high vs. low vs. control) was not significant [*F*_(1,39)_ = 2.566, *p* = 0.090, $\eta_{p}^{2}$ = 0.110]; the main effect of label types condition (hedonic labels vs. utilitarian labels) was not significant [*F*_(1,38)_ = 2.133, *p* = 0.152, $\eta_{p}^{2}$ = 0.053]; the main effect of electrode sites was significant [*F*_(1,53)_ = 8.569, *p* < 0.05, $\eta_{p}^{2}$ = 0.184].

### 700–800 ms

This was not validated by a significant social status × label types × electrode sites [*F*_(7,133)_ = 0.509, *p* = 0.826, $\eta_{p}^{2}$ = 0.026]; the main effect of status condition (high vs. low vs. control) was not significant [*F*_(1,39)_ = 0.997, *p* = 0.378, $\eta_{p}^{2}$ = 0.05]; the main effect of label types condition (hedonic labels vs. utilitarian labels) was not significant [*F*_(1,38)_ = 0.461, *p* = 0.501, $\eta_{p}^{2}$ = 0.012]; the main effect of electrode sites was significant [*F*_(2,62)_ = 8.775, *p* < 0.001, $\eta_{p}^{2}$ = 0.188].

## Discussion

This study used the Oddball paradigm to compare differences between high- and low-social-status groups in the processing of both hedonic and utilitarian labels. The results showed that in the 130–200 ms time window, the P2 effect for hedonic labels was significantly smaller than that for utilitarian labels in the low-social-status group. However, both the high-social-status group and the control group did not differ in response to different labels. This result indicated that hedonic labels were used as threatening stimuli, which affected the automatic processing trend of the low-social-status group. In the 360–450 ms time window, the P3 effect for hedonic labels was smaller than that for utilitarian labels in the low-social-status group, while the high-social-status group and control group did not differ in different labels. This suggests that individuals with a low social status have a stronger processing bias toward hedonic labels. The segmentation analysis of the 400–800 ms time window showed a dynamic trend of LPP amplitudes in low-social-status individuals at different time windows. No difference was found between the high-social-status group and the control group under different labels, which indicated that hedonic labels induced negative emotions in the low-social-status group.

The present study shows that about 130 ms after the start of the stimuli, the hedonic and utilitarian labels both induced a significant P2 component. The P2 amplitude of hedonic labels was significantly smaller than utilitarian labels only under the low-social-status condition. This result is consistent with the posed hypothesis. Previous studies have shown that the P2 amplitude in the frontal lobe area represents a rapid search for typical stimulus characteristics (Thorpe et al., 1996), and the smaller P2 amplitude represents the automated attention processing trend of an individual (Yuan et al., 2007). Therefore, the induced smaller P2 amplitude by hedonic labels indicates that the unintentional processing and preferential processing tendency of the hedonic labels can threaten individuals with a low social status. The shorter P2 latency of hedonic labels than that of utilitarian labels under low social status also indicates that individuals are faster to retrieve threat-related negative stimuli (Yuan et al., 2007). This result fully demonstrated that during the early attention processing stage, individuals with a low social status follow an automated priority processing trend for hedonic labels due to self-threat.

P3 signals the cognitive evaluation of the meaning of stimuli (Ito et al., 1998; Huang and Luo, 2006). In the present study, individuals with a low social status induced smaller P3 amplitudes under the hedonic label condition, indicating that the meaning of hedonic labels are preferentially analyzed and evaluated. Moreover, this experiment implemented the Oddball paradigm, where each stimuli presentation time was 250 ms. Therefore, the emotions of the subjects are implicitly activated in the experiment, and because the test tasks are not related to the product label stimuli, the subjects are required to inhibit their attention for irrelevant stimuli during the post-processing stage (Yuan et al., 2007). In this experimental task, both hedonic and utilitarian labels have the same inhibitions; however, in the low-social-status group, hedonic labels triggered a smaller P3 amplitude than utilitarian stimuli. This indicated that compared to utilitarian labels, hedonic labels induce more negative emotions in individuals with a low social status (Ito et al., 1998; Schupp et al., 2003; Huang and Luo, 2006). Consequently, low-social-status individuals required more inhibition for hedonic label stimuli (Smith et al., 2008; Liu et al., 2015;Morsel et al., 2017), and this difference was only found in low-social-status group, which is consistent with our initial hypothesis.

LPP is an important indicator for late emotional arousal and can dynamically reflect individual subjective emotional changes (Cuthbert et al., 2000; Mastria et al., 2017). A comparison with the LPP amplitude of the participants under 400–500, 500–600, 600–700, and 700–800 ms time windows showed that the main effect of the time window was significant, which indicated that late emotional arousal followed a dynamic changing process. The difference between the amplitudes of the 400–500 ms time window was the most significant, and the LPP amplitude of the low social status under hedonic labels was smaller than that of utilitarian labels. This indicated that hedonic labels did not carry negative emotions for the participants. However, the differences of the other three times windows were not significant, indicating that the subjects adjusted negative emotions to reduce the focus on task- irrelevant stimuli, and adjusted negative emotions that triggered smaller LPP amplitude, which was consistent with the results of the P3 volatility analysis.

The experimental results proved our expectation that different social status groups use different information processing for different types of products. The richness of social resources in the high-social-status group does not result in differences of purchase intention between both labels and they can have products with both hedonic and utilitarian attributes; therefore, no processing difference was found. However, hedonic label information will make the low-social-status group feel threatened; therefore, they will preferentially process this information. This was consistent with evolutionary studies, i.e., the human cognitive system is particularly sensitive to stimuli that generate threat perceptions (Macleod and Mathews, 1988; Mogg et al., 1998; Bar-Haim et al., 2007), and can automatically process threat stimuli as part of early cognitive processing (Huang and Luo, 2006; Franken et al., 2008). The threat is caused by an imbalance between self-evaluation and social status. The differences during the early cognitive processing stage can only prove the impact of this threat on individual cognition, but this does not indicate the way to solve this threat. This should be further explored in future research. In conclusion, this study is the first to explore the cognitive differences between high- and low-social-status groups with regard to information processing of different products. The obtained results provide a favorable basis for an in-depth analysis of decision-making and behavioral differences among different social status groups.

## Limitation

First, due to the utilized equipment, the number of participants in this study was too small, which impacted the stability of data. Second, although this study used random grouping to control the real social status of the subjects, it is unavoidable that the real social status will impact the cognitive processing process. It is therefore unclear whether the results of this study will remain valid under real social status conditions. This needs to be further explored, and research on this issue should be conducted in the future.

## Ethics Statement

Before testing, each participant signed an informed consent form. The Ethics Committee of Hunan Normal University approved this study, which monitors the ethics of research involving human subjects.

## Author Contributions

YX, WQ, and DC designed and performed the research, and analyzed the data. DC wrote the manuscript. JZ and GS proofread the manuscript and organized the materials.

## Conflict of Interest Statement

The authors declare that the research was conducted in the absence of any commercial or financial relationships that could be construed as a potential conflict of interest.
